# Environmental complexity and regularity shape the evolution of cognition

**DOI:** 10.1098/rspb.2024.1524

**Published:** 2024-10-23

**Authors:** Cameron Rouse Turner, Thomas J. H. Morgan, Thomas L. Griffiths

**Affiliations:** ^1^ Computational Cognitive Sciences Lab, Department of Computer Science, Princeton University, Princeton, NJ 08540, USA; ^2^ School of Human Evolution and Social Change, Arizona State University, Tempe, AZ 85281, USA; ^3^ Institute of Human Origins, Arizona State University, 777 E University Drive, Tempe, AZ 85287, USA

**Keywords:** information use, signal detection theory, cognitive evolution, sensory system evolution, sensory ecology

## Abstract

The environmental complexity hypothesis suggests that cognition evolves to allow animals to negotiate a complex and changing environment. By contrast, signal detection theory suggests cognition exploits environmental regularities by containing biases (e.g. to avoid dangerous predators). Therefore, two significant bodies of theory on cognitive evolution may be in tension: one foregrounds environmental complexity, the other regularity. Difficulty in reconciling these theories stems from their focus on different aspects of cognition. The environmental complexity hypothesis focuses on the reliability of sensors in the origin of cognition, while signal detection theory focuses on decision making in cognitively sophisticated animals. Here, we extend the signal detection model to examine the joint evolution of mechanisms for detecting information (sensory systems) and those that process information to produce behaviour (decision-making systems). We find that the transition to cognition can only occur if processing compensates for unreliable sensors by trading-off errors. Further, we provide an explanation for why animals with sophisticated sensory systems nonetheless disregard the reliable information it provides, by having biases for particular behaviours. Our model suggests that there is greater nuance than has been previously appreciated, and that both complexity and regularity can promote cognition.

## 1. Introduction

Even the most cognitively primitive animals are capable of gaining cues from the environment to decide which behaviour to perform. Comb jellies (*Copula sivickisi*) hunt plankton by moving towards their bioluminescent light, rather than continuing to drift [1]. Using cues remains a central function of cognition in animals with sophisticated brains capable of learning, memory and planning [2–7]. For instance, similar to comb jellies, bats (*Trachops cirrhosus*) hunt frogs (*Engystomops pustulosus*) by listening for their mating calls [8]. Nonetheless, there is little formal theory explaining the evolution of systems that detect and act on cues, and thereby make responding to information possible [9–15].

There is consensus that cognition emerges to allow animals to match behaviours to a complex and changing environment. Complexity comes in many forms: prey who change locations, or mates who vary in quality. A non-cognitive solution to complexity is to ignore information and be perpetually prepared for important contingencies, while tolerating doing worse otherwise [16]. For instance, being poisonous defends against predators without requiring information, but poison has to be produced even when predators are absent. By contrast, cognition deals with complexity by tracking and responding to change, allowing phenotypic plasticity in the form of behaviour [17–19]. We use a broad definition of cognition as the acquisition, storage, retrieval and processing of information [20]. Given that strategies that ignore information are often adequate, explaining the transition to cognition is a challenge. In response, the *environmental complexity hypothesis* posits that cognition emerges when there are multiple states that are important to the animal; further, there must be reliable cues that sufficiently covary with states, so the animal can often make correct decisions [21–35]. For instance, comb jellies evolved to hunt using bioluminescent light because (i) the location of plankton changes, and (ii) bioluminescent light reliably indicates plankton. In fact, complexity is not solely a feature of the environment, but instead arises from different outcomes of actions depending on the environment [32–36].

Although cognition requires reliable cues to be adaptive, little theory clarifies the role of sensory systems in cognitive evolution. Animals have adaptations to their sensory systems that are invested in at substantial metabolic cost (reviewed in [2–5]). For instance, nocturnal comb jellies (*C. sivickisi*) have a greater number of photoreceptors than diurnal jellies, so can hunt more effectively in the low light intensity of night [1]. Photoreceptors must confer an advantage because they are metabolically costly; a blowfly’s (*Calliphora vincia*) retina uses 8% of its energy [37]. Although cue reliability is influenced by the evolution of sensory systems, it has often been modelled as fixed [21–35,38–45]. A few models have implied that sophisticated sensors, which are reliable and metabolically costly, allow for plasticity [46–48]. However, this raises the question: how does the transition to using information occur, given that sensors are initially likely to be unreliable?

Signal detection theory suggests that environmental regularities shape how animals process information. After sensors detect cues, there is intervening processing that governs how cues produce behaviour [2–5]. A predatory bat may detect a frog, but its decision-making processes may nonetheless delay its attack until a favourable moment. Signal detection theory has provided a fruitful framework for explaining how highly consequential errors, like encountering a predator, produce biases in information processing [5,6,38–45]. Bias refers to an animal’s propensity to respond to cues so that false alarms trade-off against missed detections. For instance, small vulnerable prey should be biased to flee at the sound of a faint rustle (a weak cue), because while they will often falsely flee, they will also rarely miss detecting an actual predator. Following signal detection theory, we define *signals* to be any *cues* from a target state; this is distinct from animal communication theory, which distinguishes whether information is produced by an adaptation or inadvertently by another animal.

Prior theory suggests both environmental complexity and regularity shape cognitive evolution, but it is unclear precisely how. The environmental complexity hypothesis argues the transition to primitive cognition requires a complex environment where it is crucial to perform different actions in different situations [21–35]. By contrast, signal detection theory argues that animal cognition should have biases that often produce the same action to exploit environmental regularities (e.g. often fleeing to avoid dangerous predators [5,6,38–45]). It is unclear whether these theories are making competing claims, or if selection acts differently during the transition to cognition than it does when adapting sophisticated cognition for specific purposes. In particular, the environmental complexity hypothesis explains the evolutionary origins of information use, early nervous systems and cognitively primitive animals such as jellyfish and sea sponges, which only respond to few cues [1,49–55]. Meanwhile, signal detection theory has focused on decision making in animals with sophisticated cognition, such as zebras, chickens, frogs and crickets [56–59]. Another impediment to bridging these theories is that they are focused on different cognitive mechanisms. In particular, hypotheses about the transition to cognition emphasise sensor reliability; by contrast, work on signal detection has modelled information processing by making the simplifying assumption that sensors do not evolve and change in reliability.

Here, we extend signal detection theory to examine how sensory and information processing systems jointly underpin the evolution of cognition. While cognition naturally divides into systems that detect information and those for processing information, little theory has examined how these systems jointly evolve [2–5]. This may be because evolutionary modelling has made progress by *black-boxing* and ignoring mechanistic details [9–15]. In other cases, the general relationship between cue reliability and processing has been obscured, because the formalism is tailored to a specific scenario (i.e. communication [45,60–62], prey crypsis [63], multi-sample learning [58,64–67]). Our stylised model backgrounds many factors, such as learning, trade-offs between tasks, the animal’s internal state, and details of the precise cognitive mechanisms (e.g. for navigation). This allows us to reconcile prior theory, showing that there is greater nuance in how environmental complexity and regularity shape cognition than has previously been appreciated.

## 2. Model

Without narrowing our model’s scope, we aid understanding by describing it in terms of a prey animal who makes decisions about the presence of a predator (table 1 provides glossary and notation). Specifically, we use the escape response of the jellyfish *Aglantha digitale* as an example [51–53]. Jellyfish receive ambiguous touch cues about predators via mechanoreceptors that sometimes produce errors. Missing true signals of a predator is potentially fatal, while unnecessarily fleeing burns substantial energy through rapid swimming. While some species of jellyfish have a dedicated system for detecting predators, others cannot detect predator cues at all, indicating a transition to using information solely for escaping. After it emerged, the jellyfish predator-detection system accrued adaptations that are invested in at a metabolic cost, such as elaboration of sensory receptors and the creation of neural circuits. Selection may also alter how prey process cues by biasing the threshold for fleeing depending on cue intensity. Our mathematical model is analysed using optimality and invasion analysis (electronic supplementary material provides details).

**Table 1.** Glossary of usage and notation within the model.

	usage example	notation
**bias & discrimination**	the propensity of a reed warbler to evict a cuckoo egg for a given intensity of egg colouration is its **bias** [67]	x
	the capacity of a reed warbler to distinguish cuckoo eggs using colouration is its **discrimination**	y
**cognition & information ignoring**	a sea sponge exhibits **cognition** when it detects water current information, which is processed to release waste in strong currents [49]	∃x, y> 0
	a sea sponge that releases waste at random with respect to environmental cues is an **information ignorer**	∄x, y=0
**complexity & regularity**	a wasp inhabits a **complex environment** if its colony mates vary in dominance [68]	γ
	a wasp inhabits a **regular environment** if its colony mates are often of similar dominance	1-γ
**fastidious, impartial & gullible**	a foraging bird that approaches a snake-mimicking caterpillar only after strong evidence that it is harmless is **fastidious** [58]	x>0
	a bird that approaches on moderate evidence is **impartial**	x≈0
	a bird that approaches on weak evidence is **gullible**	x<0
**metabolic cost**	fish capable of sensing electrical disturbances produced by prey have enlarged cortices for processing electrical signals that are **metabolically costly** [69]	k, κ
**payoffs**	the expected lifetime **payoff** of successfully selecting a high-quality mate based on courtship displays will be greater when mating is infrequent	v

To provide insight into the elaboration of sensors alongside information processing, we derive the fitness of a prey animal who uses information. In particular, we consider the expected lifetime fitness of each action (fleeing or remaining) in each environmental state (a predator is present or absent). There are two types of correct detections: *hits* (flee A, predator present S) gains payoff $v_{h}$ and *correct rejections* (remain ¬A, predator absent ¬S) $v_{c}$. There are also two types of errors: *false alarms* (flee A, predator absent ¬S) lead to $v_{f}$ and *missed detections* (remain ¬A, predator present S) $v_{m}$. It is better to flee in predator presence than remain, $v_{h}>v_{m}$, but better to remain than unnecessarily flee, $v_{c}>v_{f}$. Our prey animal is scattered across an area that varies in predator density, so predators are encountered with probability $p=P\left(S\right)$. Prey observe a cue that has an intensity which is drawn from a standard Gaussian distribution centred on 0 if a predator is absent, but centred on y if a predator is present (y≥0). Here, y is the amount invested in the discrimination, such that there is an increase in the average intensity of the cue when a predator is present. While y is analogous to $d^{`}$ in classic signal detection theory, because we take y to arise from the animal’s sensory system, it is an evolving trait. Prey that observe a cue stronger than their threshold t flee. The probability of fleeing in the presence of a predator is P(A|S)=1−Φ(t−y), and in predator absence P(A|¬S)=1−Φ(t). Let Φ be the standard Gaussian cumulative distribution function. Therefore, the fitness of an information user $w_{U}$ is given by an extension of the classic Gaussian utility function of signal detection theory [70,71]. However, in addition to selection setting the prey animals' threshold for fleeing t, there is investment in discrimination, y, at metabolic cost k.


(2.1) $$w_{U}=v_{h}\text{P}\left(A|S\right)\text{P}\left(S\right)+v_{f}\text{P}\left(A|\neg S\right)\text{P}\left(\neg S\right)+v_{m}\text{P}\left(\neg A|S\right)\text{P}\left(S\right)+v_{c}\text{P}\left(\neg A|\neg S\right)\text{P}\left(\neg S\right)–ky$$

(2.2) $$w_{U}\left(t,y\right)=v_{h}\left(1-\Phi \left(t-y\right)\right)p+v_{f}\left(1-\Phi \left(t\right)\right)\left(1-p\right)+v_{m}\Phi \left(t-y\right)p+v_{c}\Phi \left(t\right)\left(1-p\right)-ky$$

Specifically, k measures the marginal loss in fitness from diverting energy to sensory systems that could be used elsewhere in the phenotype, a cost paid regardless of the outcomes of decisions.

To understand the transition to using information, we compare the fitness of an information user (∃t, y>0) with that of an information ignorer (∄t, y=0). We think of mutant information users as appearing with a poor sensor and random threshold. We assume that the fittest information ignorer adopts a blanket strategy that adapts it to withstanding predator encounters, but leaves it compromised when predators are absent: $w_{I}=v_{h}p+v_{f}\left(1-p\right)$ (as in [22]). This could represent a jellyfish that lacks touch receptors, but constantly swims briskly and thereby lessens predation. In electronic supplementary material B, we show that no generality is lost by assuming avoiding predators is paramount so that *always fleeing* is a fitter strategy than *always remaining*. Further, our conclusions are not altered by assuming that the information ignorer pays its own metabolic cost for defence (e.g. building a shell). Information users will invade when:


(2.3) $$w_{U}>w_{I}$$

Selection on information processing is easiest to understand by examining bias, rather than raw threshold. An unbiased threshold falls halfway between the expected cue intensities when a predator is absent versus present, i.e. y/2. Therefore, we transform the raw threshold t, to focus on the degree of bias using x=t-y/2, such that larger x (positive or negative) means greater bias [71]. We say that prey are impartial when x≈0, fleeing according to cue intensity. By contrast, prey are fastidious when x>0, requiring intense predator cues before fleeing. This means fewer false alarms are produced at the expense of more missed detections. Conversely, prey are gullible when x<0, more often falsely fleeing, but missing fewer detections. 'Fastidious' and 'gullible' are used technically, and without the connotation of being easily misled [5,43].

## 3. Assumptions

Our model is stylised to examine the role of environmental complexity, so it abstracts over factors known to influence cognitive evolution. First, cognitively sophisticated animals have systems that are reused for multiple tasks. While we take the standard approach of explaining our model by focusing on a single task [40,43], our model can be interpreted as describing the evolution of cognitive systems whose value is aggregated over many tasks and decisions (see electronic supplementary material C). This means we abstract over details about how reusing a cognitive system causes trade-offs between tasks, so we examine this consideration in §5. Second, when multiple decisions are made, repeated outcomes (e.g. fleeing many times) may affect animal decision-making by changing the animal’s internal state (e.g. depleting energy) [43,72–74]. We do not explicitly analyse the consequences of repeated outcomes; nonetheless, our stylised model can recover the findings of repeated outcome models (see electronic supplementary material C). Third, animals sometimes learn about signals over multiple decisions, including by making decisions to gather additional information [58,64–67]. Yet, to make initial predictions we assume no learning takes place. In particular, we assume traits are determined genetically with variance equalling one and no covariance, such that evolutionary change follows fitness gradients. However, we do not expect sensors and processing rules to strongly genetically covary and thereby threaten our conclusions (see electronic supplementary material D).

## 4. Results

**Figure 1.**
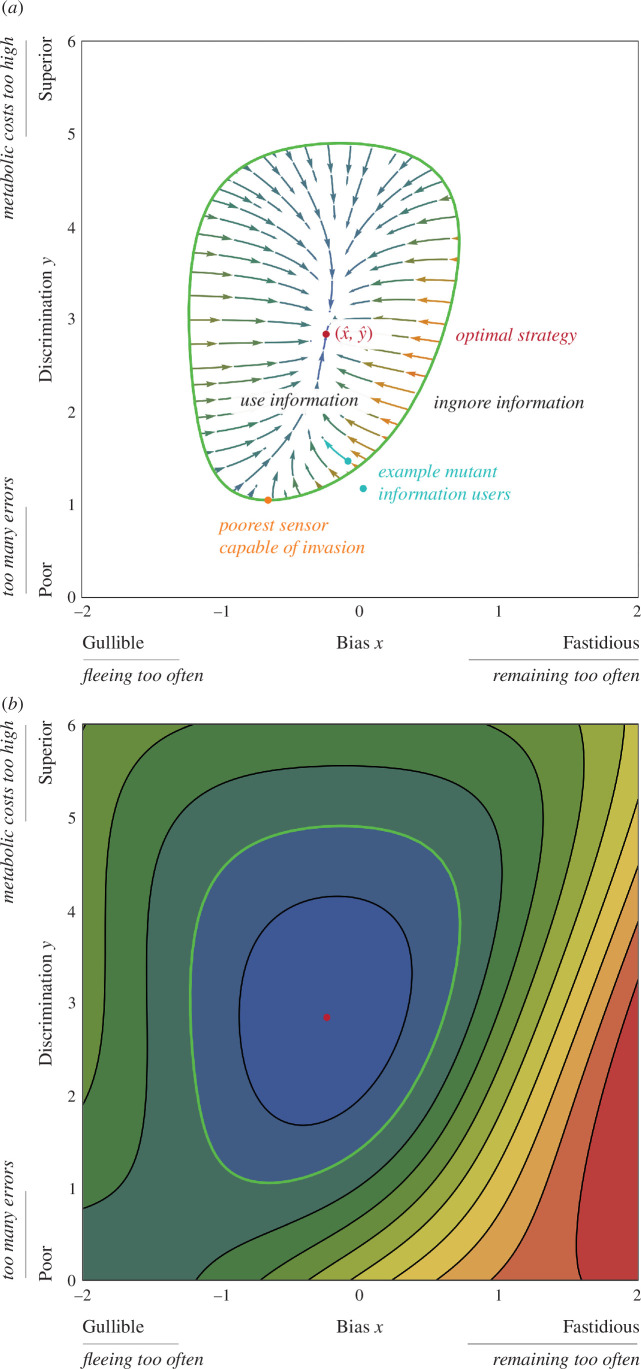
(*a*) The adaptive landscape of information use. Green boundary demarcates when a rare mutant information user can invade a population of information ignorers, so a transition to cognition occurs ($w_{U}>w_{I}$). Mutant information users likely have poor sensors (low on the *y-axis*) and random bias in processing (random on *x*-axis). Two example mutant information users are shown (teal). The mutant inside the boundary undergoes incremental cognitive elaboration towards a single stable optimum $\left(\hat{x},\hat{y}\right)$. Vectors show evolutionary dynamics (equation 4.6 and 4.7). By contrast, the mutant outside the boundary does not undergo cognitive elaboration because it is rapidly displaced by information ignorers (no directional selection on $\left(x,y\right)$). The figure assumes that avoiding predators is a greater priority than avoiding unnecessary fleeing (p = 0.25, $v_{h}=40$, $v_{m}=0$, $v_{c}=7$, $v_{f}=0.33$, k=1). (*b*) Contour plot of the adaptive landscape for information users, visualising all combinations of bias and discrimination. Information users have higher fitness as region colours become cooler.

Formally, we can understand both the transition to cognition and its subsequent elaboration by examining the adaptive landscape of information use over combinations of bias and discrimination (figure 1).

### (a) Transition to information use

#### (i) Transition condition and invasion boundary

The basic capacity to respond to cues requires producing a receptor that reacts to stimuli (e.g. photoreceptors emerged to react to light [54,55]). In jellyfish, evolving predator detection entailed linking novel touch receptors to propulsion; touch receptors apparently emerged by repurposing cellular processes that already functioned using pressure [75]. Formally, the challenge is to understand when mutants who use information can invade a population that already has an effective blanket strategy to defend against predators without requiring information. Rearranging $w_{U}>w_{I}$, we find:


(4.1) $$\left(v_{c}-v_{f}\right)\left(1-P\left(A|\neg S\right)\right)\left(1-p\right)-\left(v_{h}-v_{m}\right)P\left(\neg A|S\right)p>ky.$$

Our condition for the transition to cognition is similar to that uncovered previously but includes metabolic costs [22,23]. This result implies that the advantage of cognition is the ability to match actions to states; however, this must outweigh the vulnerability that results from errors and the expense of sensors. In context, an information-using jellyfish can conserve energy by slowing upon establishing that predators are absent, while an ignorer must perpetually swim briskly. Therefore, the conditions for information use expand when there are substantial upsides to correctly rejecting predator presence compared with falsely defending ($\left(v_{c}-v_{f}\right)\left(1-p\right)$ is large). The disadvantage of using information is that missed detections occur that leave the information user more vulnerable than information ignorers who *are* perpetually defending. This means an information-using jellyfish is selected against to the degree that meandering leaves it vulnerable when it fails to detect a predator ($\left(v_{h}-v_{m}\right)p$ is large). Crucially, the downsides of using information occur when errors are made, so cognition is favoured if actions can often be successfully matched to states ($P\left(A|\neg S\right)$ and P(¬A|S) are low). However, improving cue reliability comes at a metabolic cost (ky).

The boundary combinations of discrimination and processing that are prerequisite for information use depend solely on environmental complexity and metabolic costs. Within our model, we formally define complexity to be the degree to which both errors are similar in cost:


(4.2) $$\gamma =\frac{\left(v_{c}-v_{f}\right)\left(1-p\right)}{\left(v_{h}-v_{m}\right)p}.$$

We assumed the fittest information-ignoring strategy was to defend against predators, which entails that missed detections are the costliest error $\left(v_{h}-v_{m}\right)p>\left(v_{c}-v_{f}\right)\left(1-p\right)$. Because the parameters $\left(p,v\right)$ must obey this constraint: 0 < γ< 1. High complexity (γ→1) means jellyfish gain nearly as much fitness by avoiding unnecessary fleeing as they do detecting predators. High regularity (γ→0) means avoiding predators is paramount. Similarly, rescaled metabolic costs are defined $\kappa =\frac{k}{\left(v_{h}-v_{m}\right)p}$. Substitution into equation (4.1) shows the only parameters affecting invasion are γ and κ:


(4.3) $$\gamma \Phi \left(x+\frac{y}{2}\right)-\Phi \left(x-\frac{y}{2}\right)>\kappa y.$$

Further, substituting $1-P\left(A|\neg S\right)=\Phi \left(x+\frac{y}{2}\right)$ and $P\left(\neg A|S\right)=\Phi \left(x-\frac{y}{2}\right)$ makes clear that errors are functions of bias and discrimination. We can now easily analyse the combinations of (x,y) that allow invasion (boundary, figure 1). However, there are no closed-form solutions possible, so these were studied with parameter sweeps.

#### (ii) Regularity shapes the transition to cognition

**Figure 2.**
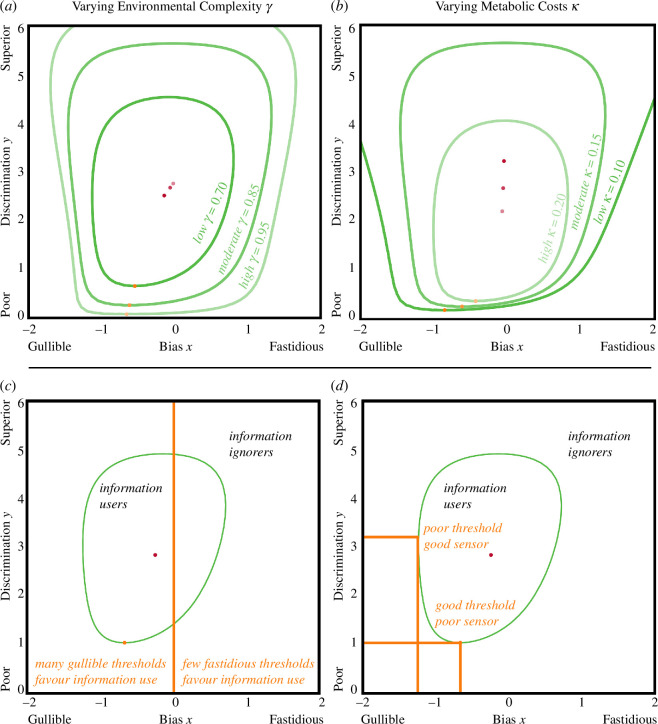
Complexity and regularity in the transition to cognition. (*a*) Greater complexity increases the boundary within which information use is favoured (unless the focal parameter γ = 0.85, κ = 0.15). (*b*) Similarly, for lower values of metabolic cost. (*c,d*) Environmental regularity constrains the transition to cognition. Regularity is due to encountering predators being more costly than falsely fleeing ($\left(v_{h}-v_{m}\right)p=10$, γ= 0.43, κ= 0.10).

Our model recovers the consensus view that complexity broadens conditions for information use [21–35], but makes explicit this is due to many combinations of sensors and bias favouring the information user (figure 2*a,b*). In particular, when complexity is high so that matching actions to states is profitable, a mutant predator-detecting jellyfish is likely to emerge because a broad range of biases and even unreliable sensors provide an advantage.

Our results also provide a caveat to the environmental complexity hypothesis, suggesting that moderate regularity does not preclude cognition but instead constrains which information-using mutants can invade. In nature, complete complexity (γ=1) is rare, with one outcome usually being more consequential than others, so realistic environments will often have moderate regularity. We find information users can still invade under realistic regularity, as long as they have biases that are responsive to important outcomes, often producing fleeing if predators are dangerous (figure 2*c*). Surprisingly, biases can compensate for poorer sensors by controlling costly errors, even though overall error rate is high (figure 2*d*). This is significant because it suggests the unreliable sensors produced by mutation may not limit the transition to cognition to the extent previously thought [21–35,46–48].

### (b) Elaboration of cognition

#### (i) Bias selected by particular errors, discrimination by aggregate errors

After primitive cue-use emerges, adaptations can arise that elaborate cognition. In the early evolution of the eye, photoreceptors not only increased in number, but their structure also changed to efficiently react to light [54,55]. Further, social paper wasps (*Polistes fuscatus*) that are capable of recognising nestmates show selection on genes underpinning both vision and central processing [68]. We provide a formal heuristic describing the joint evolution of systems for detecting and acting on cues. In particular, the analytically derived optimal bias and discrimination obeys:


(4.4) $$\hat{x}\propto D\left(\frac{\left(v_{c}-v_{f}\right)\left(1-p\right)}{k}\right)-D\left(\frac{\left(v_{h}-v_{m}\right)p}{k}\right)$$

(4.5) $$\hat{y}\propto D\left(\frac{\left(v_{c}-v_{f}\right)\left(1-p\right)}{k}\right)+D\left(\frac{\left(v_{h}-v_{m}\right)p}{k}\right)$$

Here, $D\left(\cdot \right)=\sqrt{\ln\left(\cdot /\sqrt{2\pi }\right)}$ and produces diminishing returns with regards to error costs. This is because the reduction in error rate diminishes as the extremes of the Gaussian distribution are approached (electronic supplementary material A provides derivation). As in the transition condition (equation 4.1), terms appear representing the importance of avoiding errors and correctly matching actions to states, $\left(v_{h}-v_{m}\right)p$ and $\left(v_{c}-v_{f}\right)\left(1-p\right)$. In particular, the degree of bias is a function of the *difference* between the cost of false alarms compared with missed detections. By contrast, investment in discrimination is a function of the *sum* of error costs.

While our results about bias are familiar from previous signal detection theory, we provide new insight into the role of sensor reliability. Fleeing upon weak evidence of a predator (i.e. gullibility, $\hat{x}<0$) is favoured when avoiding missed detections is important because predators are dangerous or frequent, relative to wasting energy by unnecessary fleeing ($\left(v_{h}-v_{m}\right)p$ is large). Conversely, requiring strong evidence to flee (i.e. fastidiousness, $\hat{x}>0$) is selected when avoiding false alarms is more important than missed detections ($\left(v_{c}-v_{f}\right)\left(1-p\right)$ is large). Notably, investment in superior discrimination $\hat{y}$ is favoured to the degree that both errors are costly and important to avoid. However, reducing error rates is only favourable if it provides an increase in fitness that outweighs the metabolic cost of sensors, over-and-above k. The metabolic cost of sensors affects bias, $\hat{x}\left(k\right)$, because the observer must first gain information before processing it. Our formal heuristic (equation 4.4 and 4.5) is sensible because improvements to sensory systems decrease all types of error, while bias controls one type of error at the expense of producing more of others.

#### (ii) Bias and discrimination are not always alternatives

Our model helps explain why animals who possess sophisticated sensory systems may nonetheless disregard the reliable information it provides. Intuitively, if one gains reliable information one should act in accordance with it because errors are infrequent. This implies that gaining reliable information and employing bias should be alternatives, but animals often use both tactics. For example, African savanna herbivores have keen sensors, but different species still ready themselves to flee from predators to the degree they are vulnerable [59].

**Figure 3.**
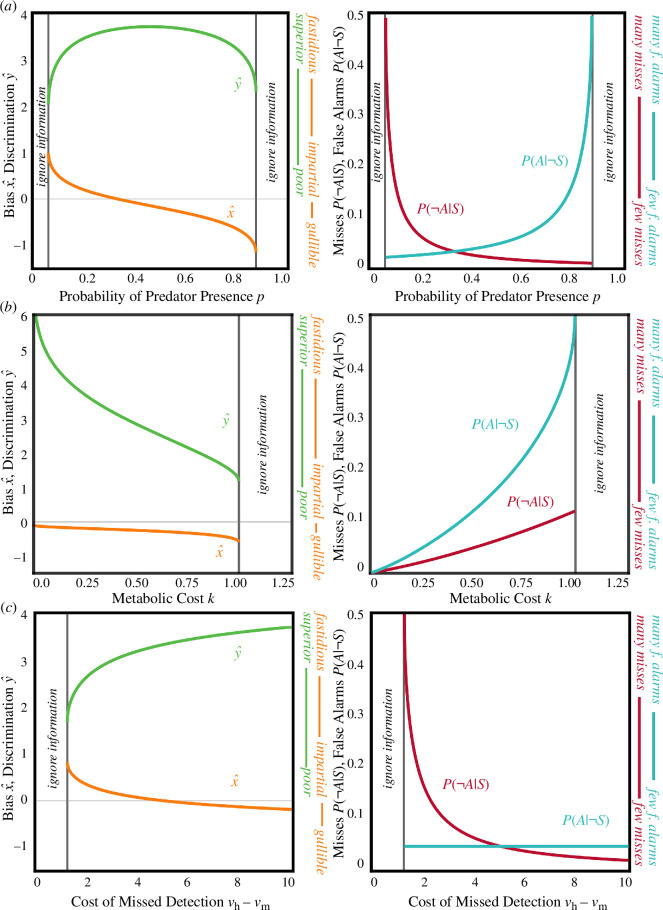
The joint evolution of bias and discrimination, and rates of errors. Unless varied (p=0.5, $v_{h}=10$, $v_{m}=0$, $v_{c}=10$, $v_{f}=5$, k = 0.25). We analysed the derivatives of $\left(\hat{x},\hat{y}\right)$ with respect to each parameter to prove that the trends depicted always hold (see electronic supplementary material F).

The intuitive case, where reliable information is heeded, occurs in highly uncertain environments or when metabolic costs are low. In highly uncertain environments (p ≈0.5) there is the chance of both frequently missing detections and falsely fleeing. This produces selection to invest in discrimination to reduce the overall error rate, alongside a propensity to heed acquired information (i.e. impartiality, figure 3*a*). Similarly, when metabolic costs are low (small k) there can be greater investment in sensors, which in turn make errors infrequent, and reduces the need for bias to control particularly costly errors (figure 3*b*).

By contrast, reliable information is partially disregarded when there is a single highly-costly outcome, such as a particularly dangerous predator (e.g. large $v_{h}-v_{m}$). If missed detections are deadly, there will be a high aggregate cost of errors, favouring sophisticated sensors (figure 3*c*). However, dangerous predators also make avoiding missed detections a greater priority than falsely fleeing. This produces strong selection on bias that overwhelms selection to heed reliable cues, so that the net effect is animals become gullible and programmatically flee. This suggests that environmental regularity, and not just complexity, can sometimes promote the elaboration of cognition, a puzzle addressed in the next section (§4c).

### (c) Elaborate or lose cognition?

Environmental regularity can lead to either the elaboration of cognitive systems or the loss of cognition altogether. Evolution frequently divests from disused cognitive systems, such as the loss of vision in animals living in the perpetual darkness of caves [2–5,76]. We found that for there to even be a transition to cognition requires a sufficiently complex environment (high γ). Yet, our results about cognitive elaboration suggest that singular highly costly errors can drive the evolution of sophisticated cognition, but this entails a regular environment that should disfavour using information at all (low γ). This apparent contradiction about the effects of regularity is resolved by considering the efficacy of preexisting cognition at the time when the environment changes to become more regular. After cognition emerges, sensory and information processing systems evolve towards the optimum $\left(\hat{x},\hat{y}\right)$ according to:


(4.6) $$\frac{\text{d}x}{\text{d}\tau }=\left(v_{c}-v_{f}\right)\left(1-p\right)\phi \left(x+\frac{y}{2}\right)-\left(v_{h}-v_{m}\right)p\phi \left(x-\frac{y}{2}\right),$$

(4.7) $$\frac{\text{d}y}{\text{d}\tau }=\frac{\left(v_{c}-v_{f}\right)}{2}\left(1-p\right)\phi \left(x+\frac{y}{2}\right)+\frac{\left(v_{h}-v_{m}\right)}{2}p\phi \left(x-\frac{y}{2}\right)-k.$$

**Figure 4.**
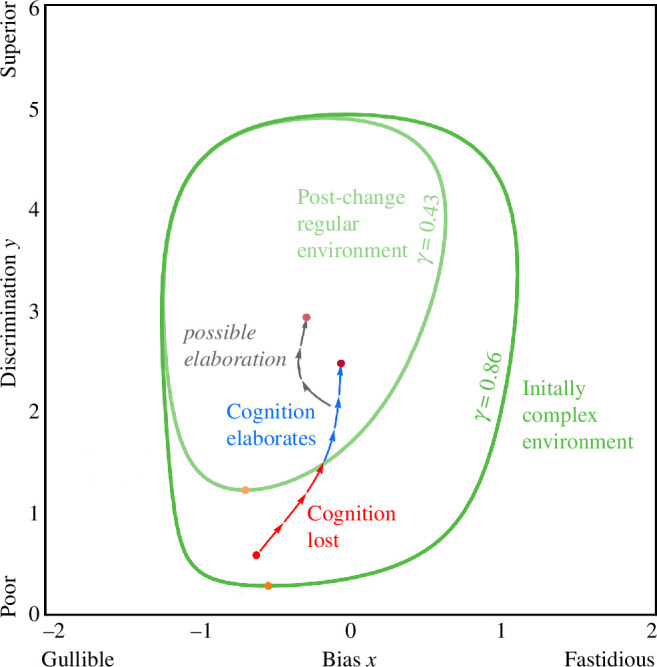
Elaboration or loss of cognition after an increase in environmental regularity. Initially, there are similar benefits of avoiding predator encounters and conserving energy (p=0.25, $v_{h}=70$, $v_{m}=35$, $v_{c}=10$, $v_{f}=0$, k =0.15). Information users invade and evolution takes the population towards an initial optimum. The environment changes so that predators become deadlier ($v_{m}=0$). Populations that have evolved to within the post-change boundary continue to elaborate (blue) and those outside are replaced by information ignorers (red). We depict an example onward cognitive elaboration trajectory (grey).

That is, how close a population gets to $\left(\hat{x},\hat{y}\right)$ depends on the amount of evolutionary time τ that has passed (see electronic supplementary material D) [77,78]. Consider an environmental change in which predators become deadlier, so the environment becomes more regular (figure 4). Such a change produces a new optimum but also reduces the conditions under which information use is favoured (shrinking the invasion boundary). Consequently, if preexisting cognition has not become sophisticated enough to sufficiently control errors, ignoring information becomes favourable and cognition is lost. Alternatively, if preexisting cognition *does* sufficiently control errors, evolution proceeds to the new optimum, which entails investing more in cognition because aggregate error costs are higher (equation 4.4 and 4.5). Remarkably, the same environmental change can lead to either the elaboration of cognition or its loss, depending on evolutionary history.

## 5. Discussion

It is unclear how two important prior bodies of theory about the evolution of cognition fit together. The transition to cognition is hypothesised to require a complex environment, alongside reliable cues [21–35]. By contrast, theory explaining decision making predicts animals will respond to important environmental regularities (e.g. readily fleeing when predators are dangerous) [5,6,38–45]. By modifying the classic signal detection model we have investigated the evolution of (i) sensors that detect cues, and (ii) information processing that dictates how cues affect behaviour. This has uncovered nuance in the ways complexity and regularity affect the transition to cognition, as well as the ultimate design of cognitive systems. Here, we discuss our model’s implications for theory about cognitive evolution. In electronic supplementary material G, we consider how our model bears on discussions of the reliability versus value of information, as well as the common simplifying assumption that observers are ideal and designed to maximise accuracy.

### (a) Predictions for cognitive evolution

Our results highlight that environmental regularity has a greater role in the transition to cognition than has been previously appreciated. Within our model, we formally defined environmental complexity as the degree to which multiple outcomes of decisions are consequential to fitness (equation 4.2). Our results affirm that complex environments support the transition to cognition, provided that sensors are metabolically cheap. However, in nature, one outcome is practically always more consequential than others (such as not finding food). This realistic regularity constrains the cognition that can emerge; evolution must wait until mutation supplies the right combinations of sensor and information processing. For example, for a predator-detecting jellyfish to have evolved from aimlessly swimming ancestors, they must have used cues to escape and meander in acceptable proportions. We find information processing can be an asset, allowing rudimentary sensors to nonetheless favour cognition by producing bias that controls particularly costly errors. However, information processing is not always valuable [23] and can hinder the transition to cognition—for instance, by producing a bias to meander too often when predators are deadly.

Our results help clarify how sensory and processing systems jointly evolve as cognition becomes sophisticated. We find that investment in sensors increases when both false alarms and missed detections are costly, while bias increases when one error is more costly than others. Events like encounters with deadly predators raise the aggregate cost of errors, favouring investment in discrimination. Concurrently, deadly predators make avoiding missed detections a high priority, favouring the evolution of biased cognition. This potentially explains why animals with sophisticated sensory adaptations nonetheless have biases that partially disregard reliable information. For example, human-specialising mosquitos have both adaptations to reliably detect human odours and a strong propensity to attack upon smelling humans [79,80]. This is apparent when compared with generalist mosquitos, who feed on various hosts from cattle to birds. Our model expects this from the fact that human populations became easier to access, becoming a particularly valuable component of the ancestral mosquito’s diet.

Our model suggests that when the environment changes to become more regular, it can either lead to cognitive elaboration or the loss of cognition altogether, depending on the preexisting efficacy of cognition. Environmental changes that make particular outcomes like predator encounters more costly can lead to divestment from cognition. This is because the environment becomes able to be negotiated with a blanket strategy that does not require information (e.g. being poisonous). However, if cognition has already become sophisticated enough to substantially control errors when the environment becomes more regular, information users can outperform information-ignoring strategies and be propelled to further elaboration. This is consistent with the broader hypothesis that animals enter a cognitive niche: once cognition becomes established it is robust to loss, increasing the chances that it is modified [81].

### (b) Further cognitive elaboration

Our stylised model made progress by backgrounding realistic details that affect the course of cognitive evolution. First, as cognition becomes sophisticated, systems are often reused for multiple tasks. Eyes are used not just to detect predators, but also to forage. It is plausible that cognition becomes adapted for multiple tasks by adding new capacities to existing architectures. For example, the first ambient blue-light photoreceptor is hypothesised to have emerged to adjust an ocean-dwelling animal’s position in the water column [54,55]. This initial photoreceptor apparently formed the basis of subsequent photoreceptors adapted to also allow directed movement, perhaps towards food. Future formal theory that unpacks details of how cognition evolved to accomplish multiple tasks will have to deal with the issue of trade-offs (a framework provided by [82]). For instance, having eyes facing forwards may improve visual acuity, which aids foraging but lessens the field of view capable of detecting predators [2–5]. Our model also abstracted over the fact that animals with sophisticated cognition adjust decision making based on changing internal states, integrating multiple cues and learning (see electronic supplementary material B & C [43,64–67,72–74]). For example, chickens (*Gallus domesticus*) increase the time they investigate an edible caterpillar if multiple cues indicate that it might be a dangerous snake [58]. Furthermore, in nature, cues often come from other animals who may evolve to obscure or facilitate information transmission [45,60–62]. Social paper wasps not only have adaptations to recognise nestmates, but nestmates have distinctive faces to make themselves recognisable [68].

## 6. Conclusion

Our model highlights that care must be taken when theorising about selection for information use. There are three connected ideas that have to be distinguished. First, there is selection to be cognitive and track the environment using correlations between events at all. Second, there is selection on sensory systems determining the quantity of information acquired upon observing the environment. Finally, there is selection on information processing that determines whether to act in accordance with cues, rather than act programmatically. The obstacle to the evolution of cognition is informational noise that inevitably leads to errors. A complex environment favours the transition to cognition because there are great upsides to producing different actions in different states, even though errors will be made. However, in nature, the outcomes of actions are never perfectly equivalent. Moderate regularities in the environment do not preclude the transition to cognition but constrain emergence to mutants whose biases compensate for their rudimentary sensors. Once cognition is established, investment in sensory systems occurs to reduce overall error rate, alongside selection on bias to avoid particularly costly errors by partially ignoring information. Once a lineage possesses sophisticated cognition that significantly controls errors, cognition becomes robust and retains value even if the environment changes to become more regular. This implies that as a lineage becomes cognitively sophisticated it also increases the likelihood of further cognitive elaboration.

## Data Availability

This article has no additional data. Supplementary material is available online [83].
